# Association Between Opioid Prescriptions and Non–US-Born Status in the US

**DOI:** 10.1001/jamanetworkopen.2020.6745

**Published:** 2020-06-02

**Authors:** Fernando A. Wilson, Kavita Mosalpuria, Jim P. Stimpson

**Affiliations:** 1 Matheson Center for Health Care Studies, The University of Utah, Salt Lake City; 2 Health Services Research and Administration, University of Nebraska Medical Center, Omaha; 3 Department of Health Management and Policy, Drexel University, Philadelphia, Pennsylvania

## Abstract

**Importance:**

Overdose from opioids causes nearly 50 000 deaths in the US each year. Adverse consequences from opioid use are particularly pronounced among low-income and publicly insured individuals. However, little is known about patterns of opioid prescribing among non–US-born individuals in the US.

**Objective:**

To examine the association of opioid prescriptions with non–US-born status, particularly among patients clinically diagnosed with pain.

**Design, Setting, and Participants:**

This cross-sectional analysis assessed opioid prescriptions among US-born and non–US-born adults using the 2016-2017 Medical Expenditure Panel Survey. Data were analyzed from January 1, 2016, to December 31, 2017.

**Main Outcomes and Measures:**

Practitioner-verified binary variable for any opioid prescription, number of prescriptions received, and a count variable for number of days of prescribed medicine. Multivariable logistic and negative binomial regression adjusted for sex, age, race/ethnicity, marital status, educational level, poverty, insurance status, clinical diagnoses for acute or chronic pain, census region, and survey year.

**Results:**

Among all 48 162 respondents (mean [SD] age, 47.0 [18.1] years; 25 831 [53.6%] female), 14.2% of US-born and 7.0% of non–US-born individuals received at least 1 opioid prescription within a 12-month period. For those diagnosed with chronic pain, 25.5% of US-born individuals and 15.6% of non–US-born individuals received at least 1 opioid prescription within a 12-month period. In multivariable logistic regression, non–US-born individuals had 35% lower odds of receiving an opioid prescription than US-born individuals (adjusted odds ratio, 0.65; 95% CI, 0.56-0.74). In negative binomial regression adjusting for confounding factors, non–US-born individuals with chronic pain who were prescribed opioids received significantly fewer days’ supply (50.0; 95% CI, 40.0-59.9) than US-born individuals (77.2; 95% CI, 72.7-81.6). Differences between US-born and non–US-born individuals were not statistically significant for patients with acute pain (16.7% [95% CI, 14.9%-18.4%] of US-born individuals received opioids vs 12.5% [95% CI, 9.3%-15.6%] of non–US-born individuals). Non–US-born individuals with less than 5 years of residency in the US were significantly less likely to receive a prescription for opioids than were those with longer residency after adjustment for type of pain and other confounding factors (adjusted odds ratio, 0.51; 95% CI, 0.30-0.88).

**Conclusions and Relevance:**

The findings suggest that non–US-born individuals, particularly those with shorter US residency, are less likely to be prescribed opioids than US-born individuals.

## Key Points

**Question:**

What are the patterns of opioid prescribing among non–US-born individuals in the US?

**Findings:**

In this cross-sectional analysis of 48 162 respondents, 14.2% of US-born and 7.0% of non–US-born individuals received at least 1 opioid prescription within a 12-month period. Non–US-born individuals with chronic pain who were prescribed opioids received significantly fewer days’ supply than US-born individuals after adjustment for confounding factors.

**Meaning:**

Non–US-born individuals may be less likely to report having pain than US-born individuals, or there may be differences in prescribing patterns among the health care professionals with prescribing authority who treat non–US-born vs US-born individuals.

## Teaser

This cross-sectional study examines the patterns of opioid prescribing among non–US-born individuals in the US.

## Introduction

The opioid epidemic continues to be a major public health threat that has been associated with high rates of overdose and heroin addiction across the US. Deaths from overdose that involve opioid use number nearly 50 000 every year.^1^ A recent study^2^ by the Society of Actuaries estimates that the opioid epidemic cost the US economy more than $630 billion from 2015 to 2018. Adverse consequences from opioid use are particularly pronounced among low-income and publicly insured individuals.^3^

The association of opioid use and overdose with race and socioeconomic status is well established. For example, 2 previous studies^3,4^ using national data on inpatient admissions and Medicare Part D data reported that patients taking opioids were more likely to reside in low-income areas. White patients are more likely than black and Hispanic patients to be hospitalized and die of prescription opioid use,^3,4^ but there is evidence that prescription opioid fatalities may be increasing more rapidly among patients in minority groups than among white patients.^5^ A study^6^ on hospital discharges associated with opioid overdose found significantly higher opioid overdose rates in low-income zip codes compared with high-income zip codes. However, little is known about the association of opioids with non–US-born status in the US. Non–US-born individuals experience high rates of poverty and often work in occupations with high risk of musculoskeletal injury, such as construction and farm industries, which are associated with high rates of opioid prescriptions.^7^ Furthermore, noncitizen populations have reduced access to care, which might be associated with opioid prescriptions.^8^ To address the knowledge gap about the prevalence of prescription opioid use in non–US-born communities, we used a large-scale, nationally representative data set, the Medical Expenditure Panel Survey (MEPS), to examine the association between opioid prescriptions and non–US-born status, particularly for those clinically diagnosed with pain.

## Methods

This cross-sectional study used the 2016-2017 MEPS, a nationally representative, in-person survey database compiled by the Agency for Healthcare Research and Quality. The MEPS was reviewed by the Westat Institutional Review Board, established under a multiproject assurance granted by the US Department of Health and Human Services Office for Protection from Research Risks. The MEPS respondents gave written consent to the US Department of Health and Human Services. For our study, the MEPS data were deidentified and publicly available and thus exempt from human subjects protocol. This study followed the Strengthening the Reporting of Observational Studies in Epidemiology (STROBE) reporting guideline.

The MEPS database provides detailed information on demographics, socioeconomic status, medical status, and prescribed medications for noninstitutionalized respondents. An important strength of MEPS is that it contacts patients’ pharmacy and other medical practitioners to help validate and augment pharmaceutical and medical records data for patients reporting that they used services. Specifically, MEPS contacts each respondent’s pharmacy practitioners by telephone and mail and requests computerized printouts of all prescriptions filled for that respondent. These data include date filled, National Drug Code, medication name, and strength and quantity dispensed. Additional information on the MEPS database is available elsewhere.^9^ Our study analyzed data from adults 18 years or older. Patients diagnosed with cancer and those younger than 18 years were excluded. Data were analyzed from January 1, 2016, to December 31, 2017.

The MEPS Prescribed Medicines files were linked to their corresponding Medical Conditions file. The Medical Conditions files provide practitioner type and clinical diagnosis codes (*International Classification of Diseases, Ninth Revision, Clinical Modification [ICD-9-CM]* and *International Statistical Classification of Diseases, Tenth Revision, Clinical Modification [ICD-10-CM]*). The primary outcomes measures were a practitioner-verified binary variable for any opioid prescription, number of prescriptions received, and a count variable for number of days of prescribed medicine across all medical settings (outpatient, office based, inpatient, and emergency department). A list of drug names classified as opioids is provided in eTable 1 in the Supplement.

The logistic regression variable was non–US-born status and defined based on US birth. Additional variables included sex, age in years, race/ethnicity (white non-Hispanic, black non-Hispanic, Hispanic, or other), married vs unmarried, educational level (less than high school, high school, or college), poverty status, insurance status (private, public, or uninsured), clinical diagnoses for chronic and acute pain, Charlson Comorbidity Index (CCI), census region, survey year, and for non–US-born individuals, length of US residency. Age was categorized as 18 to 34 years, 35 to 64 years, and 65 years or older. Poverty status was based on household incomes below 100% of the federal poverty level. The definition of the federal poverty level varies by year and family size. In 2017, for a family of 4, the federal poverty level was defined as $24 600. Clinical diagnoses of acute and chronic pain used *ICD-10-CM* codes provided by the Medical Conditions files, which were based on a crosswalk with *ICD-9-CM* codes provided by the Centers for Disease Control and Prevention (eTable 2 in the Supplement).^10^ On the basis of *ICD-10-CM* codes, the CCI adjusted for 17 comorbidities that may increase participants’ risk of mortality. The CCI was categorized as 0, 1, and 2 or higher. Length of US residency was asked of non–US-born individuals. This variable was categorized as less than 5 years of residency vs 5 or more years of residency in the US.

### Statistical Analysis

Univariate analyses included descriptive statistics stratified by non–US-born status and a comparison of receipt of any opioid prescription and number of days prescribed between US-born and non–US-born respondents. Multivariable logistic regression estimated receipt of an opioid prescription, and multivariable negative binomial regression modeled the number of days prescribed for respondents receiving any opioid prescription. These regression models were also stratified between patients diagnosed with chronic pain, acute pain, and neither chronic nor acute pain. To examine whether length of residency, defined as less than 5 years and 5 or more years, for non–US-born individuals is associated with opioid prescriptions, we used multivariate logistic regression modeling of any opioid prescription and days’ supply if prescribed for only the non–US-born population. Sensitivity and stepwise regression analyses were performed. All analyses were weighted and adjusted for complex survey design. Statistical significance was considered to be *P* < .05 using Stata software, version 16 (StataCorp).

## Results

The sample size was 48 729. After listwise deletion, the analytical sample size was 48 162, with a missing rate of 1.2%. Because of the low missing rate, imputation methods were not performed. The data set had 35 882 US-born individuals (74.5%) and 12 280 non–US-born individuals (25.5%). Among all 48 162 respondents (mean [SD] age, 47.0 [18.1] years; 25 831 [53.6%] female), 14.2% of US-born and 7.0% of non–US-born individuals received at least 1 opioid prescription within a 12-month period. For those diagnosed with chronic pain, 25.5% of US-born individuals and 15.6% of non–US-born individuals received at least 1 opioid prescription within a 12-month period. eTable 3 in the Supplement provides descriptive statistics for the number of prescriptions received and days supplied if prescribed, length of US residency, sociodemographic factors, insurance coverage, comorbidities, diagnoses of chronic and acute pain, census region, and survey year. Among individuals receiving opioid pharmaceutical treatment, US-born individuals received significantly more prescriptions (mean [SE], 3.8 [0.07]) than non–US-born individuals (mean [SE], 2.6 [0.14]). Among non–US-born individuals, 6.7% had less than 5 years of US residency. Nearly half (47.1%) of non–US-born individuals were Hispanic compared with 9.5% of US-born individuals. US-born individuals were more likely than non–US-born individuals to be 65 years or older (21.3% vs 16.3%) and less likely to be married (50.0% vs 61.8%). One in 4 non–US-born individuals had less than a high school education (24.7% vs 11.3% for US-born individuals). Non–US-born individuals also had greater likelihood of poverty (14.1% vs 10.1%) and lack of insurance (18.0% vs 6.1%) than US-born individuals. However, non–US-born individuals tended to be healthier than US-born individuals. For example, 82.0% of non–US-born individuals did not have comorbidities vs 74.7% of US-born individuals, and a lower percentage of non–US-born individuals had received a diagnosis of chronic (29.3% vs 41.0%) or acute pain (14.2% vs 23.5%) than US-born individuals.

**Figure 1.**
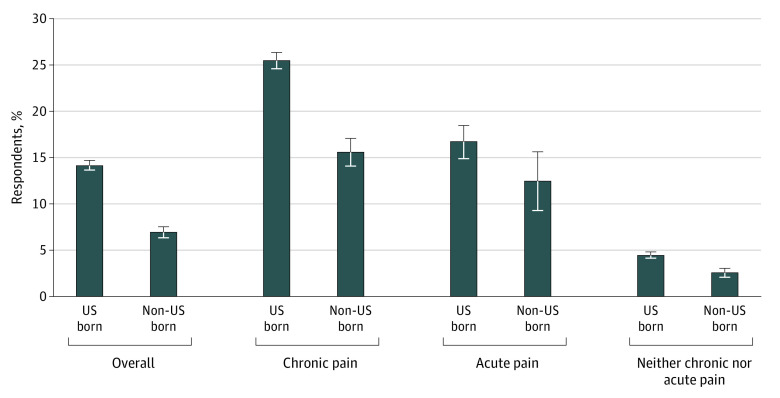
Percentage of Respondents Receiving Opioid Prescription Medications, 2016-2017 Medical Expenditure Panel Survey Error bars indicate 95% CIs.

Non–US-born individuals were significantly less likely to receive any opioid prescription within a 12-month period than US-born individuals (Figure 1). Among all respondents, 7.0% of non–US-born individuals received an opioid prescription compared with 14.2% of US-born individuals. For non–US-born individuals clinically diagnosed with chronic pain, 15.6% received an opioid prescription compared with 25.5% of US-born individuals (difference, 9.9%). Among respondents with a diagnosis of acute pain only, 16.7% (95% CI, 14.9%-18.4%) of US-born individuals received opioids compared with 12.5% (95% CI, 9.3%-15.6%) of non–US-born individuals, although differences for acute pain were not statistically significant. Furthermore, a small percentage of respondents received opioid prescriptions for diagnoses unrelated to acute or chronic pain, accounting for 4.5% of US-born and 2.6% of non–US-born individuals.

**Figure 2.**
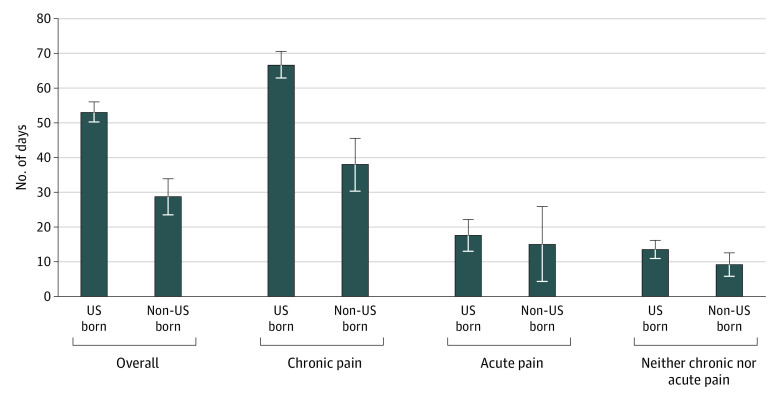
Mean Number of Days’ Supply of Opioid Prescription Medications for Respondents Receiving an Opioid Prescription, 2016-2017 Medical Expenditure Panel Survey Error bars indicate 95% CIs.

As shown in Figure 2, after receiving an opioid prescription, non–US-born individuals generally received significantly fewer days of treatment than US-born individuals. For all respondents with a prescription, non–US-born individuals received a supply of 28.7 days compared with 53.1 days for US-born individuals. Patients with chronic pain born in the US received an additional 28.6 days of opioid medications compared with non–US-born individuals with chronic pain (66.6 vs 38.0 days). US-born patients with acute pain (17.7 [95% CI, 13.3-22.0] for US-born patients and 15.2 [95% CI, 4.5-25.8] for non–US-born patients) or neither chronic nor acute pain (13.6 [95% CI, 11.0-16.3] for US-born patients and 9.4 [95% CI, 6.0-12.8] for non–US-born patients) received more days of supply than non–US-born individuals, but these differences were not statistically significant.

In multivariable regression of receipt of any opioid prescription, non–US-born individuals had 35.0% lower adjusted odds of receiving any opioid prescription than US-born individuals (adjusted odds ratio [AOR], 0.65; 95% CI, 0.56-0.74) (Table 1). Results were similar for patients diagnosed with chronic pain (AOR, 0.64; 95% CI, 0.54-0.75). Although non–US-born individuals with acute pain had lower adjusted odds of receiving an opioid prescription than US-born individuals, this result was not statistically significant (AOR, 0.68; 95% CI, 0.47-1.0). However, among individuals with neither chronic nor acute pain, non–US-born individuals had significantly lower adjusted odds of receiving an opioid prescription than US-born individuals (AOR, 0.69; 95% CI, 0.52-0.92).

**Table 1.** Multivariable Logistic Regression Adjusted Odds Ratios of Any Opioid Prescription Use, 2016-2017 Medical Expenditure Panel Survey^a^

Variable	Adjusted odds ratio (95% CI)			
	Overall (N = 48 162)	Pain		
		Chronic (n = 17 850)	Acute (n = 3477)	None (n = 26 835)
US born	1 [Reference]	1 [Reference]	1 [Reference]	1 [Reference]
Foreign born	0.65 (0.56-0.74)	0.64 (0.54-0.75)	0.68 (0.47-1.0)	0.69 (0.52-0.92)
Sex				
Female	1 [Reference]	1 [Reference]	1 [Reference]	1 [Reference]
Male	0.80 (0.74-0.86)	0.87 (0.79-0.95)	0.97 (0.77-1.22)	0.55 (0.46-0.65)
Race/ethnicity				
Non-Hispanic white	1 [Reference]	1 [Reference]	1 [Reference]	1 [Reference]
Non-Hispanic black	0.94 (0.85-1.04)	1.03 (0.91-1.16)	0.91 (0.65-1.28)	0.78 (0.62-0.96)
Hispanic	0.85 (0.75-0.96)	0.85 (0.73-0.99)	1.0 (0.71-1.41)	0.81 (0.62-1.05)
Other	0.82 (0.69-0.96)	0.74 (0.61-0.90)	1.14 (0.69-1.88)	0.86 (0.62-1.17)
Age group, y				
18-34	1 [Reference]	1 [Reference]	1 [Reference]	1 [Reference]
35-64	1.16 (1.04-1.29)	1.30 (1.13-1.51)	1.33 (1.0-1.78)	0.80 (0.65-0.98)
≥65	0.95 (0.84-1.08)	1.05 (0.89-1.24)	0.91 (0.62-1.32)	0.83 (0.63-1.09)
Married				
No	1 [Reference]	1 [Reference]	1 [Reference]	1 [Reference]
Yes	1.05 (0.97-1.14)	0.98 (0.89-1.08)	1.01 (0.79-1.30)	1.27 (1.06-1.53)
Educational level				
Less than high school	1 [Reference]	1 [Reference]	1 [Reference]	1 [Reference]
High school	0.96 (0.86-1.08)	0.92 (0.80-1.08)	1.02 (0.70-1.48)	1.10 (0.83-1.45)
College	0.81 (0.72-0.90)	0.70 (0.61-0.80)	0.84 (0.59-1.20)	1.21 (0.92-1.59)
Below poverty level				
No	1 [Reference]	1 [Reference]	1 [Reference]	1 [Reference]
Yes	1.11 (1.0-1.24)	1.12 (0.99-1.28)	1.07 (0.72-1.58)	1.10 (0.87-1.39)
Insurance coverage				
Private	1 [Reference]	1 [Reference]	1 [Reference]	1 [Reference]
Public	1.33 (1.22-1.47)	1.35 (1.21-1.51)	1.40 (1.03-1.92)	1.21 (0.97-1.49)
Uninsured	0.67 (0.56-0.80)	0.75 (0.59-0.94)	0.73 (0.42-1.25)	0.59 (0.42-0.82)
Charlson Comorbidity Index				
0	1 [Reference]	1 [Reference]	1 [Reference]	1 [Reference]
1	1.35 (1.18-1.55)	1.26 (1.08-1.47)	1.44 (0.90-2.31)	1.75 (1.24-2.47)
≥2	1.77 (1.61-1.94)	1.65 (1.48-1.83)	0.98 (0.72-1.34)	2.88 (2.29-3.62)
Diagnosis of chronic pain				
No	1 [Reference]	NA	NA	NA
Yes	3.39 (3.11-3.69)	NA	NA	NA
Diagnosis of acute pain				
No	1 [Reference]	1 [Reference]	NA	NA
Yes	3.17 (2.93-3.44)	2.83 (2.58-3.09)	NA	NA

Abbreviation: NA, not applicable.

^a^ Regressions adjusted for all variables in table and survey year. Odds ratios were adjusted for complex survey design of the Medical Expenditure Panel Survey.

Among respondents receiving an opioid prescription, multivariable negative binomial regression modeled the incidence rate ratio (IRR) of number of days’ supply of opioid prescriptions. The IRR for non–US-born vs US-born respondents was 0.75 (95% CI, 0.63-0.90) among all patients (eTable 4 in the Supplement). In negative binomial regression, US-born individuals received a supply of opioids for 52.7 days (95% CI, 49.9-55.5 days) compared with 39.5 days (95% CI, 33.0-46.0) for non–US-born individuals after adjustment for confounding factors. After stratifying by type of pain, US-born patients with chronic pain received more than 2 months’ supply of opioids (77.2 days; 95% CI, 72.7-81.6 days) compared with a supply for non–US-born individuals with chronic pain of 50.0 days (95% CI, 40.0-59.9 days). Results on days’ supply were not statistically significant between non–US-born individuals and US-born individuals with acute pain (78.3 days [95% CI, 12.7-43.8 days] vs 18.6 days [95% CI, 15.7-21.5 days]) and patients with neither chronic nor acute pain (15.8 days [95% CI, 10.8-20.8 days] vs 15.2 days [95% CI, 13.2-17.2 days]).

Additional multivariable regressions examined the association of length of US residency in years among non–US-born individuals with receiving an opioid prescription (Table 2). Non–US-born individuals with less than 5 years of residency in the US were significantly less likely to receive a prescription for opioids than were those with longer residency after adjustment for type of pain and other confounding factors (AOR, 0.51; 95% CI, 0.30-0.88). Results for length of residency were not statistically significant for days’ supply.

**Table 2.** Multivariable Regression Adjusted Odds Ratios and Incidence Rate Ratios of Opioid Prescription Use for Foreign-Born Individuals, 2016-2017 Medical Expenditure Panel Survey^a^

Variable	Odds ratio of receiving an opioid prescription (95% CI) (n = 13 607)	Incidence rate ratio of days’ supply (95% CI) (n = 1362)
Length of US residency, y		
≥5	1 [Reference]	1 [Reference]
<5	0.51 (0.30-0.88)	1.28 (0.47-3.46)
Sex		
Female	1 [Reference]	1 [Reference]
Male	0.68 (0.55-0.83)	1.10 (0.83-1.46)
Race/ethnicity		
Non-Hispanic white	1 [Reference]	1 [Reference]
Non-Hispanic black	0.96 (0.62-1.47)	0.86 (0.52-1.42)
Hispanic	0.80 (0.58-1.12)	0.83 (0.59-1.17)
Other	0.58 (0.41-0.82)	0.64 (0.44-0.94)
Age group, y		
18-34	1 [Reference]	1 [Reference]
35-64	0.96 (0.72-1.28)	1.52 (1.06-2.20)
≥65	0.95 (0.66-1.37)	2.87 (1.84-4.48)
Married		
No	1 [Reference]	1 [Reference]
Yes	1.14 (0.91-1.42)	0.82 (0.62-1.08)
Educational level		
Less than high school	1 [Reference]	1 [Reference]
High school	1.05 (0.81-1.36)	0.93 (0.64-1.34)
College	0.99 (0.78-1.27)	0.55 (0.39-0.78)
Below poverty level		
No	1 [Reference]	1 [Reference]
Yes	1.03 (0.80-1.33)	0.70 (0.52-0.94)
Insurance coverage		
Private	1 [Reference]	1 [Reference]
Public	0.88 (0.69-1.12)	1.20 (0.87-1.65)
Uninsured	0.48 (0.33-0.69)	0.67 (0.41-1.09)
Charlson Comorbidity Index		
0	1 [Reference]	1 [Reference]
1	1.90 (1.25-2.88)	1.47 (0.95-2.29)
≥2	1.88 (1.41-2.52)	1.76 (1.35-2.31)
Diagnosis of chronic pain		
No	1 [Reference]	1 [Reference]
Yes	2.95 (2.34-3.73)	1.87 (1.37-2.55)
Diagnosis of acute pain		
No	1 [Reference]	1 [Reference]
Yes	3.84 (3.12-4.74)	1.34 (1.0-1.79)

^a^ Regressions adjusted for all variables in table and survey year. Odds ratios were adjusted for complex survey design of the Medical Expenditure Panel Survey.

We also stratified the analyses by health care setting and examined differences in opioid prescriptions by non–US-born status (eTable 5 in the Supplement). Health care settings included office-based physician, outpatient, emergency department, inpatient, and dental offices. Approximately half of opioid prescriptions were from office-based physicians, but there were no statistically significant differences in practitioner setting by non–US-born status. As additional sensitivity analysis, we excluded opioid prescriptions associated with dental services, but results were not substantively different for non–US-born vs US-born status in the receipt of any opioid prescription (AOR, 0.64; 95% CI, 0.56-0.74) or for number of days’ supply (IRR, 0.74; 95% CI, 0.61-0.88).

We compared unadjusted differences in the receipt of opioid prescriptions by non–US-born status with differences adjusted for socioeconomic status, access to care, and comorbidities (eTable 6 in the Supplement). Although differences between US- and non–US-born individuals decreased with these additional factors, there were no statistically significant differences across AORs.

We examined interactions in the probability of receiving opioid prescriptions and non–US-born status with chronic pain, acute pain, health insurance, poverty status, and age (eTable 7 in the Supplement). In every case, non–US-born individuals had lower likelihood of receiving an opioid prescription compared with US-born individuals, although the difference was not statistically significant for individuals with no acute pain. For example, an estimated 12.4% (95% CI, 11.5%-13.3%) of US-born individuals with public insurance received an opioid prescription compared with 6.9% (95% CI, 5.8%-8.0%) of non–US-born individuals. Likelihood of opioid prescriptions was not significantly different across racial/ethnic groups among US-born individuals. However, white non-Hispanic, non–US-born individuals were estimated to have significantly higher likelihood of opioid prescriptions (8.3%; 95% CI, 6.2%-10.4%) than Hispanic (5.5%; 95% CI, 4.9%-6.2%) or Asian (4.9%; 95% CI, 3.9%-5.8%) non–US-born individuals.

## Discussion

The opioid epidemic shows few signs of abating, with overdose deaths associated with opioid use increasing every year since 2012.^1^ Despite the important implications of the epidemic for public health, little is known about its population health consequences for non–US-born communities. We used nationally representative data on sociodemographic factors, medical conditions, and prescriptions to examine the association of opioid use with non–US-born status in the US. Non–US-born individuals were significantly less likely to receive a prescription for opioid medications than US-born individuals regardless of whether they were clinically diagnosed with pain. For example, although 1 in 4 US-born individuals diagnosed with chronic pain were prescribed opioids, only 15% of non–US-born individuals received an opioid prescription. Furthermore, among patients receiving prescriptions for opioids, non–US-born patients were generally treated for a substantially shorter period than US-born patients. US-born individuals with diagnosed chronic pain received more than 70 days’ supply of opioids in contrast to the 44-day supply for non–US-born individuals with chronic pain. However, we did not find statistically significant differences in prescriptions for opioids among patients diagnosed with only acute pain. In addition, our data did not show statistically significant differences in the source of opioid prescriptions across practitioner settings (office-based physician, outpatient, emergency department, inpatient, and dental) between US and non–US-born respondents. Interacted regression analysis showed that white non-Hispanic, non–US-born individuals had a significantly higher likelihood of receiving opioid prescriptions than did Hispanic or Asian non–US-born individuals. Differences in opioid prescriptions were not significantly different across racial/ethnic groups for US-born individuals.

Reasons for differences in prescribing of opioids between non–US-born and US-born individuals are unclear and warrant further research. One interpretation of our findings is that non–US-born individuals may be less likely to report experiencing pain than US-born individuals. However, we adjusted for clinical diagnosis of chronic and acute pain using *ICD-10-CM* codes, which mitigates this possibility. Another possible explanation for the differences is the specialties of practitioners with prescribing authority that treat non–US-born vs US-born individuals. For example, a recent article^11^ reported that 22.3% of dental prescriptions were for opioids, such as oxycodone, for patients of US dentists compared with 0.6% of prescriptions written by dentists in England. Prior research^12^ found that non–US-born individuals were significantly less likely to use dental services than US-born individuals. However, our sensitivity analysis suggested that differences in opioid prescriptions by non–US-born status remained after opioid prescriptions associated with dental services were excluded. Other research using Medicare Part D prescription claims data indicates that nurse practitioners and physician assistants accounted for three-quarters of prescribers having the highest 5% of opioid prescription proportions.^13^ Furthermore, most high rates of opioid prescribing were concentrated in urgent care clinics.^13^ In our data, US-born individuals are more likely to report a nurse practitioner or physician assistant as their usual source of care (4.5%) than non–US-born individuals (1.1%). Other research has demonstrated that non–US-born individuals also use urgent care settings, such as emergency departments, at lower rates than US-born individuals.^14,15,16^ In addition, there have been long-standing issues with racial/ethnic disparities in quality of care in the treatment of pain, which may help explain the differences observed in our data.^17,18^ Prior literature has documented inadequate pain management for racial/ethnic minority groups even after adjustment for demographic factors and pain intensity.^18^ However, an important implication of the lower likelihood of opioid prescribing among non–US-born individuals in our study is that it may have helped protect non–US-born communities from the high rates of opioid-related mortality occurring in the US.^19^

It is also unclear whether non–US-born individuals are more likely to receive non–pharmaceutical-based therapies for pain if they are less likely to be prescribed opioid medications than US-born individuals. Further research is needed to explore this hypothesis. Furthermore, our analysis found that non–US-born individuals with less than 5 years of residency in the US were less likely to receive a prescription for opioids than those with longer residency. Our analysis adjusted for access to care measures, such as poverty and uninsured status. However, recently immigrated individuals experience significant legal barriers to qualify for federally funded benefits programs. Under the Personal Responsibility and Work Opportunities Act of 1996,^8^ undocumented immigrants and most authorized immigrants are ineligible for Medicaid, Medicare, and other benefit programs unless funding is provided by the state. As a result, compared with longer-residency non–US-born individuals, non–US-born individuals with shorter residency may have different characteristics of insurance coverage, types of practitioners seen, or access to care difficulties associated with the likelihood of receiving a prescription.

### Limitations

This study has limitations. Although MEPS attempts to validate medical care use of respondents, respondents may have recall bias on using any medical care in the prior 12-month period. However, it is unclear whether this recall bias systematically differs between US-born and non–US-born individuals. The non–US-born individuals may also be less likely to participate in surveys than US-born respondents, which may affect representativeness of the MEPS. In addition, we do not have data on whether prescribed opioid use resulted in differing rates of opioid misuse or abuse between US-born and non–US-born individuals. Differences in temporal trends in opioid prescribing are unclear and warrant further research. Also, the sample of non–US-born respondents reporting acute pain is limited in size.

## Conclusions

Our findings suggest that non–US-born individuals are significantly less likely to receive an opioid prescription than US-born individuals, particularly among those with diagnosed chronic pain. Furthermore, non–US-born individuals receiving opioid prescriptions had fewer days’ supply of prescriptions than US-born individuals after adjustment for chronic and acute pain and other confounding factors. Moreover, shorter length of residency for non–US-born individuals was associated with a lower rate of opioid prescriptions. Differences in opioid prescribing remained after adjustment for confounding factors and with interactions for key variables. Reasons for these differences are unclear and warrant further research.

## Notes

Supplement. **eTable 1.** List of Drug Names Classified as Prescription Opioids **eTable 2.** Categorization of ICD-9-CM and ICD-10-CM Codes by Acute and Chronic Pain **eTable 3.** Descriptive Statistics in Weighted Percent (95% CI) Stratified by Non–US-Born Status, MEPS 2016-2017 **eTable 4.** Multivariable Negative Binomial Regression-Adjusted Incidence Rate Ratios (IRR) (95%CI) of number of Days of Opioid Prescription Use, MEPS 2016-2017 **eTable 5.** Distribution of Number of Opioid Prescriptions If Received an Opioid Prescription by Healthcare Setting (Percent [95% CI]) Stratified by Non–US-Born Status, MEPS 2016-2017 **eTable 6.** Multivariable Logistic Regression-Adjusted Odds Ratios (AOR) (95% CI) of Any Opioid Prescription Use, MEPS 2016-2017 **eTable 7.** Multivariable Logistic Regression-Adjusted Probabilities of Any Opioid Prescription (95% CI) With Interactions, MEPS 2016-2017
