# Supplementary material for: Association Between Opioid Prescriptions and Non–US-Born Status in the US
Source: JAMA Netw Open. 2020 Jun 2;3(6):e206745. doi: 10.1001/jamanetworkopen.2020.6745 (PMC7267847; doi:10.1001/jamanetworkopen.2020.6745)
Supplement: Supplement. — eTable 1. List of Drug Names Classified as Prescription Opioids eTable 2. Categorization of ICD-9-CM and ICD-10-CM Codes by Acute and Chronic Pain eTable 3. Descriptive Statistics in Weighted Percent (95% CI) Stratified by Non–US-Born Status, MEPS 2016-2017 eTable 4. Multivariable Negative Binomial Regression-Adjusted Incidence Rate Ratios (IRR) (95%CI) of number of Days of Opioid Prescription Use, MEPS 2016-2017 eTable 5. Distribution of Number of Opioid Prescriptions If Received an Opioid Prescription by Healthcare Setting (Percent [95% CI]) Stratified by Non–US-Born Status, MEPS 2016-2017 eTable 6. Multivariable Logistic Regression-Adjusted Odds Ratios (AOR) (95% CI) of Any Opioid Prescription Use, MEPS 2016-2017 eTable 7. Multivariable Logistic Regression-Adjusted Probabilities of Any Opioid Prescription (95% CI) With Interactions, MEPS 2016-2017 [file jamanetwopen-3-e206745-s001.pdf]

Supplementary Online Content

Wilson FA, Mosalpuria K, Stimpson JP. Association between opioid prescriptions and non–US-born status in the US. *JAMA Netw Open*. 2020;3(6):e206745. doi:10.1001/jamanetworkopen.2020.6745

eTable 1. List of Drug Names Classified as Prescription Opioids

eTable 2. Categorization of ICD-9-CM and ICD-10-CM Codes by Acute and Chronic Pain

eTable 3. Descriptive Statistics in Weighted Percent (95% CI) Stratified by Non–US-Born Status, MEPS 2016-2017

eTable 4. Multivariable Negative Binomial Regression-Adjusted Incidence Rate Ratios (IRR) (95%CI) of number of Days of Opioid Prescription Use, MEPS 2016-2017

eTable 5. Distribution of Number of Opioid Prescriptions If Received an Opioid Prescription by Healthcare Setting (Percent [95% CI]) Stratified by Non–US-Born Status, MEPS 2016-2017

eTable 6. Multivariable Logistic Regression-Adjusted Odds Ratios (AOR) (95% CI) of Any Opioid Prescription Use, MEPS 2016-2017

eTable 7. Multivariable Logistic Regression-Adjusted Probabilities of Any Opioid Prescription (95% CI) With Interactions, MEPS 2016-2017

This supplementary material has been provided by the authors to give readers additional information about their work.

© 2020 Wilson FA et al. *JAMA Network Open.*

eTable 1. List of Drug Names Classified as Prescription Opioids

Drug Name
Codeine and combinations
Atropine-Diphenoxylate
Buprenorphine
Butorphanol
Fentanyl
Hydrocodone and combinations
Hydromorphon
Meperidine
Methadone
Morphine
Nalbuphine
Oxycodone
Oxycontin
Oxymorphone
Pentazocine
Tramadol

© 2020 Wilson FA et al. *JAMA Network Open.*

eTable 2. Categorization of ICD-9-CM and ICD-10-CM Codes by Acute and Chronic Pain*

Description	ICD-9-CM Codes	ICD-10-CM
Acute Pain from Disease	282 338 522 574 577 592 733 800 801 802 803 804 805 806 807 808 809 810 811 812 813 814 815 816 817 818 819 820 821 822 823 824 825 826 827 828 829 830 831 832 833 834 835 836 837 838 839 840 841 842 843 844 845 846 847 848 850 851 852 853 854 860 861 862 863 864 865 866 867 868 869 870 871 872 873 874 875 876 877 878 879 880 881 882 883 884 885 886 887 890 891 892 893 894 895 896 897 900 901 902 903 904 910 911 912 913 914 915 916 917 918 919 920 921 922 923 924 925 926 927 928 929 930 931 932 933 934 935 936 937 938 939 940 941 942 943 944 945 946 947 948 949 950 951 952 953 954 955 956 957 958 959	D55 D56 D57 D58 G89 K04 K80 K85 K86 L08 M48 M80 M81 M84 M85 M87 M89 M94 N20 R52 S00 S01 S02 S03 S04 S05 S06 S07 S08 S09 S10 S11 S12 S13 S14 S15 S17 S19 S20 S21 S22 S23 S24 S25 S26 S27 S28 S29 S30 S31 S32 S33 S34 S35 S36 S37 S38 S39 S40 S41 S42 S43 S44 S45 S46 S47 S48 S49 S50 S51 S52 S53 S54 S55 S56 S57 S58 S59 S60 S61 S62 S63 S64 S65 S66 S67 S68 S69 S70 S71 S73 S72 S73 S74 S75 S76 S77 S78 S79 S80 S81 S82 S83 S84 S85 S86 S87 S88 S89 S90 S91 S92 S93 S94 S95 S96 S97 S98 S99 T07 T14 T15 T16 T17 T18 T19 T20 T21 T22 T23 T24 T25 T26 T27 T28 T30 T31 T32 T79
Chronic Pain	338 346 307 710 711 712 713 714 715 716 717 718 719 720 721 722 723 724 725 726 727 728 729	G43 G44 G89 M00 M01 M02 M05 M06 M08 M11 M12 M13 M14 M15 M16 M17 M18 M19 M20 M23 M24 M25 M32 M33 M34 M35 M36 M43 M45 M46 M47 M48 M49 M50 M51 M53 M54 M60 M61 M62 M65 M66 M67 M70 M71 M72 M75 M76 M77 M79 M96 R25 R26 R29 R52

ICD, International Classification of Diseases; CM, Clinical Modification

* Categorization follows Centers for Disease Control and Prevention’s “Guide to ICD-9-CM and ICD-10 Codes Related to Poisoning and Pain”.

© 2020 Wilson FA et al. *JAMA Network Open.*

eTable 3. Descriptive Statistics in Weighted Percent (95% CI) Stratified by Non–US-Born Status, MEPS 2016-2017*

	Overall (n = 48,162)	US-born (n = 35,882)	Foreign-born (n = 12,280)
Length of US residency			
>= 5 years	N.A.	N.A.	93.3 (92.7, 93.9)
< 5 years	N.A.	N.A.	6.7 (6.1, 7.3)
Days supplied if prescribed, number	50.8 (48.1, 53.5)	53.1 (50.1, 56.0)	28.7 (23.5, 33.9)
Prescriptions received if prescribed, number	3.7 (3.6, 3.8)	3.8 (3.7, 4.0)	2.6 (2.4, 2.9)
Male	48.2 (47.7, 48.8)	48.2 (47.6, 48.9)	48.2 (47.1, 49.4)
Race/ethnicity			
White, non-Hispanic	63.3 (62.8, 63.8)	73.1 (72.6, 73.6)	16.2 (15.0, 17.3)
Black, non-Hispanic	11.8 (11.5, 12.1)	12.7 (12.3, 13.0)	7.8 (7.3, 8.4)
Hispanic	15.9 (15.6, 16.3)	9.5 (9.2, 9.8)	47.1 (45.9, 48.2)
Other	8.9 (8.6, 9.2)	4.7 (4.5, 5.0)	28.9 (27.8, 30.0)
Age			
18-34	29.8 (29.2, 30.3)	30.6 (30.0, 31.2)	25.5 (24.5, 26.6)
35-64	49.8 (49.2, 50.4)	48.1 (47.4, 48.7)	58.1 (57.0, 59.3)
65 and over	20.4 (20.0, 20.9)	21.3 (20.8, 21.8)	16.3 (15.5, 17.2)
Married	52.0 (51.5, 52.6)	50.0 (49.4, 50.7)	61.8 (60.6, 62.9)
Education			
Less than high school	13.6 (13.2, 13.9)	11.3 (10.9, 11.7)	24.7 (23.8, 25.6)
High school	29.0 (28.5, 29.5)	30.2 (30.0, 30.7)	23.5 (22.6, 24.5)
College	57.4 (56.8, 58.0)	58.6 (57.9, 59.2)	51.8 (50.6, 52.9)
In poverty	10.8 (10.5, 11.1)	10.1 (9.7, 10.4)	14.1 (13.4, 14.8)
Insurance coverage			
Private	70.6 (70.1, 71.1)	73.1 (72.6, 73.6)	58.4 (57.2, 59.5)
Public	21.3 (20.8, 21.7)	20.8 (20.3, 21.3)	23.6 (22.7, 24.5)
Uninsured	8.2 (7.9, 8.5)	6.1 (5.8, 6.4)	18.0 (17.2, 18.8)
Charlson Co-morbidity Index			
0	76.0 (75.5, 76.5)	74.7 (74.2, 75.3)	82.0 (81.2, 82.9)
1	6.6 (6.3, 6.9)	7.2 (6.8, 7.5)	3.8 (3.8, 4.2)
2 or higher	17.4 (17.0, 17.9)	18.1 (17.6, 18.6)	14.2 (13.4, 15.0)
Diagnosis of:			
Chronic pain	39.0 (38.5, 39.6)	41.0 (40.4, 41.7)	29.3 (28.2, 30.4)
Acute pain	21.9 (21.4, 22.3)	23.5 (22.9, 24.0)	14.2 (13.4, 15.1)
Neither chronic or acute	52.8 (52.2, 53.4)	50.3 (49.6, 50.9)	65.1 (64.0, 66.2)
Census region			
1	17.8 (17.3, 18.3)	17.3 (16.8, 17.8)	20.2 (19.2, 21.2)
2	20.9 (20.4, 21.3)	22.9 (22.4, 23.5)	11.0 (10.3, 11.7)
3	37.6 (37.1, 38.2)	38.3 (37.6, 38.9)	34.6 (33.5, 35.7)
4	23.7 (23.2, 24.2)	21.5 (21.0, 22.0)	34.3 (33.2, 35.3)

© 2020 Wilson FA et al. *JAMA Network Open.*

Survey year			
2016	49.8 (49.2, 50.3)	49.8 (49.1, 50.4)	49.7 (48.5, 50.9)
2017	50.2 (49.7, 50.8)	50.2 (49.6, 50.9)	50.3 (49.1, 51.5)

CI, Confidence Interval; MEPS, Medical Expenditure Panel Survey; N.A., Not Applicable

© 2020 Wilson FA et al. *JAMA Network Open.*

eTable 4. Multivariable Negative Binomial Regression-Adjusted Incidence Rate Ratios (IRR) (95%CI) of number of Days of Opioid Prescription Use, MEPS 2016-2017*

	Overall (n = 5,257)	Chronic Pain (n = 3,418)	Acute Pain (n = 403)
US born	Ref.	Ref.	Ref.
Foreign born	0.75 (0.63, 0.90)	0.65 (0.52, 0.80)	1.52 (0.87, 2.66)
Sex			
Female	Ref.	Ref.	Ref.
Male	1.09 (0.99, 1.20)	1.12 (1.0, 1.25)	1.03 (0.76, 1.39)
Race/ethnicity			
White, non-Hispanic	Ref.	Ref.	Ref.
Black, non-Hispanic	0.93 (0.82, 1.04)	0.91 (0.80, 1.04)	0.44 (0.31, 0.63)
Hispanic	0.78 (0.65, 0.92)	0.84 (0.68, 1.02)	0.51 (0.31, 0.84)
Other	0.84 (0.65, 1.07)	0.89 (0.69, 1.14)	0.31 (0.18, 0.54)
Age			
18-34	Ref.	Ref.	Ref.
35-64	2.0 (1.70, 2.36)	2.40 (1.89, 3.04)	1.90 (1.40, 2.58)
65 and over	2.26 (1.89, 2.71)	2.70 (2.10, 3.48)	1.65 (1.06, 2.58)
Married			
No	Ref.	Ref.	Ref.
Yes	0.96 (0.86, 1.06)	0.95 (0.85, 1.07)	1.11 (0.83, 1.49)
Education			
Less than high school	Ref.	Ref.	Ref.
High school	1.01 (0.89, 1.16)	1.03 (0.89, 1.19)	1.0 (0.64, 1.56)
College	0.87 (0.76, 0.99)	0.88 (0.77, 1.02)	.93 (0.60, 1.45)
In poverty			
No	Ref.	Ref.	Ref.
Yes	1.10 (0.97, 1.24)	1.15 (1.02, 1.30)	0.84 (0.46, 1.50)
Insurance coverage			
Private	Ref.	Ref.	Ref.
Public	1.67 (1.50, 1.88)	1.64 (1.46, 1.83)	2.20 (1.36, 3.55)
Uninsured	1.21 (0.97, 1.51)	1.22 (0.93, 1.60)	1.55 (1.04, 2.30)
Charlson Co-morbidity Index			
0	Ref.	Ref.	Ref.
1	1.25 (1.06, 1.47)	1.29 (1.07, 1.55)	.93 (0.60, 1.46)
2 or higher	1.38 (1.24, 1.53)	1.42 (1.26, 1.59)	1.51 (1.0, 2.26)
Diagnosis of chronic pain			
No	Ref.		
Yes	2.89 (2.51, 3.32)		
Diagnosis of acute pain			
No	Ref.	Ref.	
Yes	1.08 (0.98, 1.19)	1.03 (0.93, 1.16)	

IRR, Incidence Rate Ratio; MEPS, Medical Expenditure Panel Survey; CI, Confidence Interval

© 2020 Wilson FA et al. *JAMA Network Open.*

* Regressions adjust for all variables in table and survey year. IRRs are adjusted for complex survey design of MEPS.

© 2020 Wilson FA et al. *JAMA Network Open.*

eTable 5. Distribution of Number of Opioid Prescriptions If Received an Opioid Prescription by Healthcare Setting (Percent (95% CI)) Stratified by Non–US-Born Status, MEPS 2016-2017*

	Overall (n = 5,950)	US-born (n = 4,968)	Foreign-born (n = 982)
Office-based physician	48.6 (46.8, 50.4)	48.7 (46.8, 50.6)	47.5 (42.5, 52.6)
Outpatient	12.1 (10.9, 13.3)	12.9 (11.6, 14.2)	16.8 (12.5, 21.2)
Emergency Room	13.3 (12.1, 14.4)	12.1 (10.9, 13.4)	11.7 (8.3, 15.1)
Inpatient	13.3 (12.0, 14.5)	13.3 (12.0, 14.5)	13.3 (10.3, 16.4)
Dental office	12.8 (11.5, 14.0)	13.0 (11.6, 14.4)	10.6 (7.6, 13.5)

CI, Confidence Interval; MEPS, Medical Expenditure Panel Survey

© 2020 Wilson FA et al. *JAMA Network Open.*

eTable 6. Multivariable Logistic Regression-Adjusted Odds Ratios (AOR) (95% CI) of Any Opioid Prescription Use, MEPS 2016-2017*

	Overall (n = 48,162)	Chronic Pain (n = 17,850)	Acute Pain (n = 3,477)	Neither Chronic nor Acute (n = 26,835)
Model 1	0.46 (0.42, 0.51)	0.56 (0.49, 0.64)	0.73 (0.53, 1.0)	0.58 (0.46, 0.72)
Model 2	0.52 (0.45, 0.59)	0.60 (0.51, 0.70)	0.68 (0.47, .98)	0.64 (0.48, 0.86)
Model 3	0.57 (0.50, 0.65)	0.62 (0.53, 0.73)	0.68 (0.47, .99)	0.69 (0.52, 0.92)

CI, Confidence Interval; AOR, Adjusted Odds Ratio; MEPS, Medical Expenditure Panel Survey

Model 1 adjusts for survey year and region.
Model 2 adds sex, race/ethnicity, age, marital status, and education.
Model 3 adds poverty status, insurance status, and Charlson Co-Morbidity Index.

© 2020 Wilson FA et al. *JAMA Network Open.*

eTable 7. Multivariable Logistic Regression-Adjusted Probabilities of Any Opioid Prescription (95% CI) With Interactions, MEPS 2016-2017*

Interactions with foreign-born status	Probability (95%CI)
Chronic pain	
Yes	
US-born	18.4 (17.5, 19.3)
Foreign-born	12.6 (11.0, 14.2)
No	
US-born	6.2 (5.8, 6.6)
Foreign-born	4.2 (3.4, 4.9)
Acute pain	
Yes	
US-born	13.9 (12.8, 15.1)
Foreign-born	10.7 (8.9, 12.5)
No	
US-born	5.0 (4.6, 5.3)
Foreign-born	3.0 (2.5, 3.5)
Health insurance	
Private insurance	
US-born	9.3 (8.8, 9.8)
Foreign-born	7.2 (6.2, 8.3)
Public insurance	
US-born	12.4 (11.5, 13.3)
Foreign-born	6.9 (5.8, 8.0)
No insurance	
US-born	7.0 (5.8, 8.3)
Foreign-born	3.2 (2.2, 4.2)
Poverty status	
Yes	
US-born	10.8 (9.8, 11.9)
Foreign-born	5.8 (4.6, 7.0)
No	
US-born	9.5 (9.1, 10.0)
Foreign-born	6.6 (5.8, 7.5)
Age group	
18-34	
US-born	9.1 (8.3, 9.9)
Foreign-born	6.3 (4.9, 7.8)
35-64	
US-born	10.5 (9.9, 11.1)
Foreign-born	6.8 (5.8, 7.8)
65 and over	
US-born	8.7 (7.9, 9.4)
Foreign-born	6.0 (4.8, 7.1)
Race/ethnicity	

© 2020 Wilson FA et al. *JAMA Network Open.*

White, non-Hispanic	
US-born	10.0 (9.5, 10.6)
Foreign-born	8.3 (6.2, 10.4)
Black non-Hispanic	
US-born	9.5 (8.7, 10.3)
Foreign-born	7.1 (5.1, 9.2)
Hispanic	
US-born	9.0 (8.0, 10.1)
Foreign-born	5.5 (4.9, 6.2)
Asian	
US-born	9.2 (7.7, 10.8)
Foreign-born	4.9 (3.9, 5.8)

CI, Confidence Interval; MEPS, Medical Expenditure Panel Survey

© 2020 Wilson FA et al. *JAMA Network Open.*
